# Unique Presentation of Idiopathic Retroperitoneal Fibrosis in a Primary Care Setting

**DOI:** 10.7759/cureus.18429

**Published:** 2021-10-01

**Authors:** Salam Khalil, Nerosanth Selvarajah, Satish Solanki, Holli Neiman-Hart, Glenn Dregansky

**Affiliations:** 1 Family and Community Medicine, Western Michigan University Homer Stryker M.D. School of Medicine, Kalamazoo, USA; 2 Internal Medicine, Western Michigan University Homer Stryker M.D. School of Medicine, Kalamazoo, USA

**Keywords:** idiopathic, retroperitoneal, fibrosis, fibro-inflammatory, periaortitis, hydronephrosis

## Abstract

Patients with retroperitoneal fibrosis (RPF), a rare condition, present with nonspecific abdominal pain or flank pain that can be complicated by urologic obstruction and/or vascular compromise. Reporting rare entities that often elude prompt diagnosis will aid clinicians to consider the entity in their differential diagnosis and potentially lead to earlier recognition and appropriate management. Our case emphasizes the importance of not just the diagnosis and treatment of RPF but how to best monitor RPF patients to minimize complications from disease progression or treatment-related adverse events.

## Introduction

Retroperitoneal fibrosis (RPF) is a rare disease with an incidence of 0.1-1.3 cases per 100,000 persons per year [1]. It is characterized by fibro-inflammatory changes that affect soft tissues in proximity to the distal abdominal aorta [1]. It commonly includes tissues in the retroperitoneal space such as the ureters and common iliac arteries [2].

The disease is more commonly seen in males than females with a ratio ranging between 2:1 and 3:1, with a mean age of onset of approximately 55 and 60 years [3]. Over 75% of cases are considered idiopathic, but a substantial number of cases can be due to secondary causes such as medications, autoimmune diseases, malignancies, and other etiologies [4].

The clinical presentation of RPF depends on the impact of fibrotic changes on the surrounding tissues [5,6]. The most common presentation includes unilateral or bilateral flank pain, lower abdominal pain, and body fatigue. The pain can be colicky depending on partial or complete obstruction of the ureters.

RPF is diagnosed primarily based on computed tomography (CT) or magnetic resonance imaging (MRI) [1]. Usually, a biopsy is not required for the diagnosis; however, it may be needed in situations such as atypical location of the fibrosis, suspected malignancy or infection, mass extending above the renal arteries, or anterior displacement of the aorta [7].

Although the primary treatment for RPF is medical management, surgical procedures may be required for immediate relief of ureteral or vascular obstructions. Glucocorticoids and immunosuppressants are the mainstays of medical management [8,9]. However, in the case of ureteral involvement, immediate relief of obstruction is the first goal [1]. The relief can be accomplished by ureterolysis or, more conservatively, stent placement [4].

## Case presentation

A 45-year-old female patient presented initially to the primary care clinic with symptoms of dyspnea, cough, and wheezing along with anterior abdominal wall tenderness. She had a medical history significant for bilateral venous insufficiency, smoking, obesity, and bipolar type II disorders. The patient tested negative for coronavirus disease 2019 and was sent home with a provisional diagnosis of viral respiratory infection. Her condition showed mild improvement with conservative supportive treatment.

**Figure 1:**
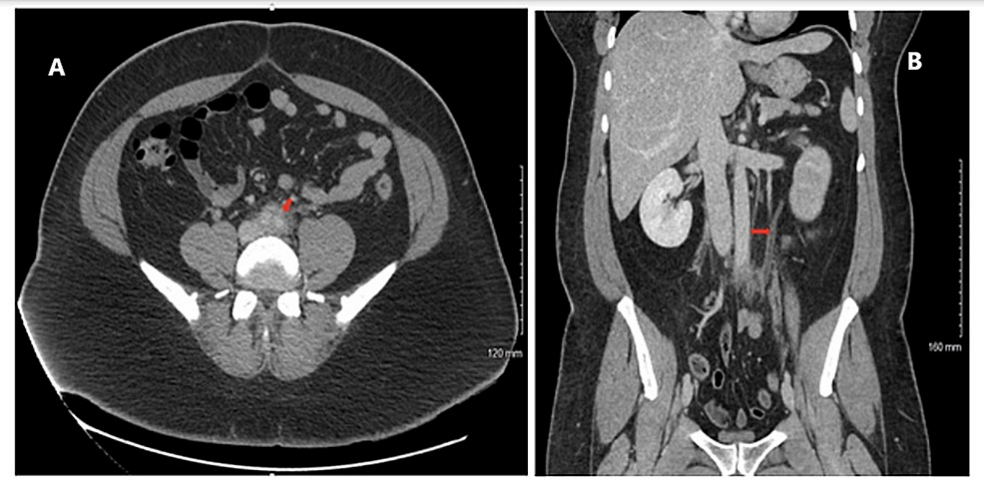
CT abdomen and pelvis with contrast. The red arrow in A shows retroperitoneal fibrosis surrounding the descending vessels originating from the abdominal aorta. The red arrow in B shows left ureteral hydronephrosis before stent placement. CT: computed tomography

After four months, the patient presented to the emergency department with complaints of epigastric abdominal pain, nausea, vomiting, and diarrhea for the past month. Initial laboratory workup in the emergency department was significant for an elevated creatinine of 1.42 mg/dL, C-reactive protein (CRP) of 6.2 mg/L, and an erythrocyte sedimentation rate (ESR) of 41 mm/hour. The polymerase chain reaction test for severe acute respiratory syndrome coronavirus 2 was negative. Before diagnostic imaging, a 2 L bolus of normal saline fluid was administered intravenously followed by maintenance fluid. CT scan of the abdomen and pelvis with contrast was significant for RPF, periaortitis involving the distal aorta and its bilateral common iliac arteries, decreased perfusion of the left kidney with mild hydronephrosis, and obstruction of a portion of the left distal ureter (Figure 1). A high-resolution chest CT scan reported multiple small areas of clustered cystic and/or emphysematous changes scattered throughout the lungs and a few ground-glass nodules in the right lung apex.

**Figure 2:**
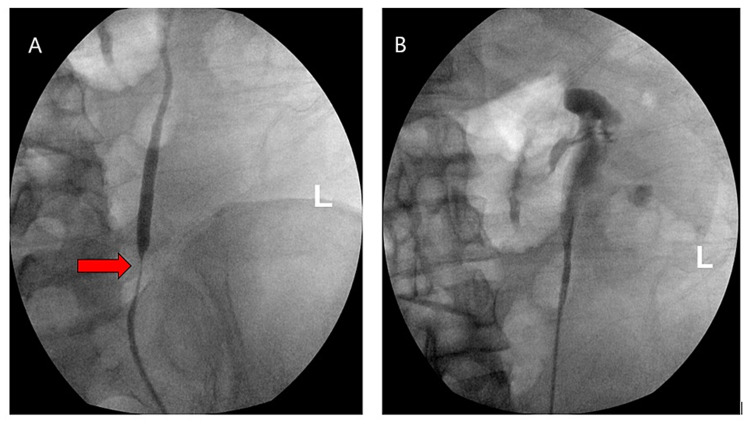
X-ray urography with left retrograde pyelogram before (A) and after stent placement (B). The red arrow in A indicates the stricture of the left ureter noted at the distal third region.

Interventional radiology was consulted for a percutaneous biopsy of retroperitoneal fibrotic tissue but it was not completed due to a safe window being inaccessible. In addition, urology was consulted due to left hydronephrosis and for intra-ureteral biopsy. X-ray urography with left retrograde pyelogram showed stricture of the left ureter at the distal third region. Therefore, a stent was placed in the left ureter (Figure 2). During the procedure, a biopsy could not be obtained due to extrinsic compression of the ureter without intrinsic lesions.

During her hospital course, an extensive autoimmune workup was completed to investigate secondary causes of RPF. Apart from positive antinuclear antibody test with a titer of 1:160, all other investigations were within normal limits, including C-antineutrophil cytoplasmic antibodies (ANCA) or PR3-ANCA, P-ANCA or MP3-ANCA, anti-smooth muscle antibodies, anti-thyroglobulin antibodies serum protein electrophoresis with reflex to immunofix, and immunoglobulin subclass immunoglobulin G4. Due to the lack of secondary causes, the patient was diagnosed with idiopathic retroperitoneal fibrosis (IRPF) and was discharged on 40 mg of prednisone once daily with close rheumatology and primary care follow-up.

At the primary care follow-up visit one week after hospital discharge, the patient was initially diagnosed with new-onset type 2 diabetes mellitus with a plan to taper steroid and initiate metformin. At her four-week rheumatology follow-up, the patient was started on disease-modifying anti-rheumatic drugs (DMARDs), mycophenolate 500 mg twice daily, and bridged with continued moderate-dose prednisone. ESR, CRP, and creatinine were followed up and eventually normalized. The patient underwent a bilateral renal ultrasound which showed resolution of hydronephrosis after four weeks of treatment. The patient visited the emergency department for uncontrolled diabetes and was diagnosed with diabetic ketoacidosis (DKA), which required inpatient treatment. Her DKA state quickly resolved within the first 24 hours after starting insulin drip and discharged home on an insulin regimen consisting of long-acting (detemir) 25 units twice a day and a short-acting (aspart) sliding scale. However, the patient had low C-peptide (1.1 ng/mL) which prompted a change in her diagnosis to new-onset type 1 diabetes mellitus. The patient continued to follow up with the primary care physician every week to monitor control of her blood glucose. The patient also followed up with urology, and her left ureteral stent was removed in three months.

## Discussion

IRPF, formerly known as Ormond’s disease, is a very uncommon condition with the typical age of onset ranging between 40 and 60 years. The pathology develops with fibro-inflammatory changes in the retroperitoneal space, and it may affect the abdominal aorta, common iliac arteries, the inferior vena cava, and the ureters. To date, there are no guidelines on the management of IRPF. Secondary etiologies attributed to genetics, immunology, malignancy, and atherosclerosis should be taken into consideration irrespective of patient presentation.

The presentation of the disease can vary from subtle symptoms to more acute emergent symptoms requiring emergency treatment. IRPF should be suspected in patients complaining of flank or abdominal pain, arterial insufficiency, urinary tract obstruction, and newly detected kidney impairment. Our patient presented with severe flank and abdominal pain, and, subsequently, renal impairment.

Imaging with MRI or CT and biopsy can be used to reach the diagnosis of RPF. In some cases, interventional radiology may not be able to obtain a biopsy due to the unpredictable nature of the fibrosis, either due to its location, involvement, or severity. In our case, imaging was the sole diagnostic test of choice and was sufficient to reach the diagnosis. However, biopsy is recommended when the location of the fibro-inflammatory changes is atypical and not periaortic or peri-iliac, or high clinical suspicion for malignancy or infection exists, or if the diagnosis is not obtainable via radiologic images [7].

In cases of IRPF, symptom relief should be the utmost priority to ensure stopping or slowing the progression of the disease, reducing obstructive symptoms, and preventing relapse. A multi-specialty approach along with close monitoring for complications by primary care physicians is crucial in the initial stages of management of IRPF. The most common pharmaceutical interventions include steroid or immunosuppressant therapy. Corticosteroid therapies lead to a reduction in the inflammatory response and potentially a quick improvement of symptoms. The initial dose of 40-60 mg of prednisone or 1 mg/kg/day once daily is initiated, and the duration of therapy can last from months to years. In addition to corticosteroids, DMARDs such as mycophenolate mofetil, methotrexate, and rituximab can be used to stabilize the disease and promote regression [8,9]. Our patient initially responded well to the steroid therapy, but soon developed hyperglycemia and peripheral neuropathy. The combination of steroid and immunosuppressant drugs resulted in an additive effect leading to severe hyperglycemia that caused an emergency visit. When such changes are made, although the therapy’s responsiveness can be unpredictable, the primary care physician should closely follow up to monitor complications.

Our patient’s HbA1c reduced from 5.2-6.5% to 10.7% in approximately three months, which is likely attributed to the onset and progression of disease along with aggressive disease management. Mycophenolate therapy alone has been associated with new-onset hyperglycemia or worsening of pre-existing diabetes [10]. However, when combined with corticosteroids, it may further contribute to the high metabolic and hyperglycemic adverse effects [11]. Therefore, clinicians should monitor serum glucose levels routinely. The development of type 1 diabetes mellitus in our patient was most likely secondary to the underlying IRPF, given that our patient had a low C-peptide level which supports a lack of insulin production by the pancreas [12].

Aside from case reports, case series, and other nonclinical trial studies, there are no specific guidelines on the use of immunosuppressive and biological agents regarding the treatment of IRPF. There is some evidence that suggests that the use of other agents such as azathioprine, cyclophosphamide, methotrexate, and rituximab has been beneficial when used in individuals who have failed glucocorticoid therapy. There are no preferred second- or third-line agents for the treatment of IRPF. However, mycophenolate and methotrexate are the most commonly used agents based solely on the frequency of use [13].

## Conclusions

IRPF is a unique fibro-inflammatory progressive disease with a variety of systemic presentations. Imaging or biopsy is needed to confirm the diagnosis. Patients should be started on immunosuppressive therapy immediately to control their condition. Surgical intervention may be considered to relieve symptoms. Our case re-emphasizes the unpredictable nature of IRPF and the significance of the primary care physician’s involvement.
